# Use of PEG-asparaginase in newly diagnosed adults with standard-risk acute lymphoblastic leukemia compared with *E. coli*-asparaginase: a retrospective single-center study

**DOI:** 10.1038/srep39463

**Published:** 2016-12-21

**Authors:** Wen-jian Liu, Hua Wang, Wei-da Wang, Meng-yuan Zhu, Cheng-cheng Liu, Jing-hua Wang, Yue Lu

**Affiliations:** 1 State Key Laboratory of Oncology in South China, Collaborative Innovation Center for Cancer Medicine, Guangzhou, Guangdong, 510060, People’s Republic of China; 2 Department of Hematologic Oncology, Sun Yat-sen University Cancer Center, Guangzhou, Guangdong, 510060, People’s Republic of China

## Abstract

Acute lymphoblastic leukemia (ALL) is a heterogeneous disease, and the long-term survival varies with different ages. We performed a retrospective analysis of 122 newly diagnosed adults with standard-risk ALL treated with *Escherichia coli* asparaginase (*E. coli*-asparaginase, n = 50) and polyethylene glycol-conjugated asparaginase (PEG-asparaginase, n = 72). No treatment-related mortality (TRM) occurred in the *E. coli*-asparaginase group, and 3 TRM events occurred in the PEG-asparaginase group without relation to asparaginase. In addition, 22 (44.0%) and 48 (66.7%) patients achieved a complete response (CR) on day 14 in the *E. coli*-asparaginase and PEG-asparaginase groups, respectively (*P* = 0.032). No different 5-year event-free survival (EFS) or overall survival (OS) rate (*P* = 0.632 and 0.769) was observed. Multivariate analysis revealed later CR (*P* = 0.008) and older age (*P* = 0.049) as adverse prognostic factors for both EFS and OS. In addition, we specifically monitored the known adverse effects of asparaginase, and no asparaginase-related death was observed. Allergy occurred in 9 patients using *E. coli*-asparaginase, and no patient in the PEG-asparaginase group suffered from allergies (*P* < 0.001). The incidence of other asparaginase-related toxicities was similar. We conclude that PEG-asparaginase can be safely and effectively used as asparaginase in adults with newly diagnosed standard-risk ALL.

Acute lymphoblastic leukemia (ALL) is a heterogeneous disease, and outcomes vary by age, immunophenotype, and cytogenetic and molecular features. Asparaginase, a bacterial enzyme that depletes serum asparagine, is a standard component in most combination chemotherapy protocols for ALL^1^ both in children^2^,^3^,^4^,^5^,^6^ and adults^7^,^8^. ALL cells lack asparagine synthetase and are dependent on the plasma levels of this amino acid for survival^9^,^10^. Plasma asparagine depletion leads to inhibition of protein synthesis, which results in inhibition of nucleotide synthesis and subsequent apoptotic cell death of the leukemic cells^1^,^11^. Three types of asparaginase are currently available; these asparaginases are derived from two different bacterial sources: *Escherichia coli* and *Erwinia chrysanthemi*^12^,^13^,^14^. Native *Escherichia coli* asparaginase (*E. coli*-asparaginase) and polyethylene glycol-conjugated asparaginase (PEG-asparaginase) are both derived from *Escherichia coli,* whereas *Erwinia* asparaginase is derived from *Erwinia chrysanthemi*^12^,^13^,^14^. The most commonly used form of asparaginase is *E. coli*-asparaginase^14^.

A major limitation of *E. coli*-asparaginase is the development of hypersensitivity, which is reported in 15 to 73% of cases^12^,^14^,^15^,^16^. Other side effects, such as thrombosis, pancreatitis, hyperglycemia, hepatotoxicity, and abnormalities of lipid metabolism, also contribute to the limitations^12^,^17^,^18^. Compared with *E. coli*-asparaginase, PEG-asparaginase produces prolonged depletion of asparagine and is associated with reduced incidence of certain toxicities (i.e., hypersensitivity reactions), thereby making it preferable for use in ALL treatment^19^,^20^,^21^.

The development of treatment for children has improved with each successive study, with long-term survival achieved in more than 80% of patients^22^,^23^,^24^. Similarly, steady improvements in the cure rate for adults have been achieved through accurate diagnoses; the use of intensive combination chemotherapy; attention to potential sanctuary sites, such as the central nervous system (CNS); and the appropriate use of allogeneic hematopoietic stem-cell transplant (allo-HSCT). However, the long overall survival (OS) and event-free survival (EFS) of ALL in adults remain poor compared with children, and no clear consensus has been reached as to whether allo-HSCT is advantageous compared with the most effective available chemotherapy for consolidation of adults with standard-risk ALL while in the first complete response (CR1)^25^,^26^,^27^.

Since 2005, adults in our center with standard-risk ALL, as defined according to the criteria of the United Kingdom Medical Research Council Adult Leukemia Working Party and Eastern Cooperative Oncology Group (MRC UKALL XII/ECOG) E2993 protocol^28^,^29^, have received induction, intensification, consolidation, and maintenance therapy using the MRC UKALL XII/ECOG E2993-based regimen, including *E. coli*-asparaginase or PEG-asparaginase as asparaginase (Table 1). However, allo-HSCT was not recommended after CR1. Given that limited information has been reported on the comparison between *E. coli*-asparaginase and PEG-asparaginase in adults with newly diagnosed ALL to date, we investigated this issue in our single-center series.

## Results

### Patient characteristics

From January 2005 to January 2013, 59 patients first received *E. coli*-asparaginase, and 9 patients were excluded due to allergy to *E. coli*-asparaginase and switched to *Erwinia* asparaginase. In addition, none of the 9 patients experienced a subsequent allergic event while receiving *Erwinia* asparaginase. In total, 72 patients received PEG-asparaginase, and no patients suffered from allergy. Thus, a total of 122 patients with standard-risk ALL were included. The median age in the *E. coli*-asparaginase and PEG-asparaginase groups was 27 years (range 18–35) and 26 years (range 18–35), respectively (P = 0.822). Correspondingly, the median white blood cell (WBC) of the two groups was 26.25 (0.50–94.06) × 10^9^/L and 26.92 (1.08–99.74) × 10^9^/L, with no difference between the two groups (P = 0.379). The baseline characteristics for patients receiving *E. coli*-asparaginase and PEG-asparaginase regimens are presented in Table 2. No significant differences were observed between the two groups with respect to age, sex, immunophenotype, Eastern Cooperative Oncology Group (ECOG) performance status, extramedullary leukemia, and cerebrospinal fluid (CSF) positivity at diagnosis.

### Toxicity and clinical management

Asparaginase-related toxic events, including pancreatitis, transaminitis, hyperbilirubinemia, hypoalbuminemia, hyperglycemia, hypertriglyceridemia, hypofibrinogenemia and deep vein thrombosis (DVT), were comparable and without statistical differences (Table 3). No asparaginase-related toxic deaths were observed in either group.

All patients suffered from grade IV myelosuppression, which included the lowest WBC counts of 0.05–0.89 × 10^9^/L (median 0.42 × 10^9^/L) and 0.03–0.96 × 10^9^/L (median 0.39 × 10^9^/L), hemoglobin level of 49–63 g/L (median 57 g/L) and 52–61 g/L (median 56 g/L), and platelet count of 2–24 × 10^9^/L (median 13 × 10^9^/L) and 4–22 (median 13 × 10^9^/L) in the two groups, respectively. Other non-specific toxicities, including hair loss, nausea and vomiting, oral ulcers, fever, bacteremia, and the use of intravenous antifungal therapy for suspected invasive fungal disease, were similar for both groups (data not shown).

No treatment-related mortality (TRM) occurred in the *E. coli*-asparaginase group. In contrast, 3 TRM events occurred in the PEG-asparaginase group without relation to asparaginase. The 3 deaths were considered to be directly related to grade 4 myelosuppression and sepsis in the consolidation therapy (2 in cycle 1 and 1 in cycle 3).

Grade 3/4 pancreatitis was managed with delay asparaginase, alimentation, analgesia, octreotide, and antibiotics. These measures are effective when initiated upon the first symptoms and signs of acute pancreatitis. Moreover, the asparaginase treatment was delayed but not reduced in dose when grade 3/4 hepatotoxicity developed. Careful monitoring was conducted when toxicity resolves to grade 1/2, and cryoprecipitate infusion was administered when serum fibrinogen was less than 50 mg/dL. Patients suffering from thrombotic complications were anticoagulated with low-molecular-weight heparin and subsequent warfarin without terminating the use of asparaginase.

### Response, survival, relapse and follow-up treatment

**Figure 1:**
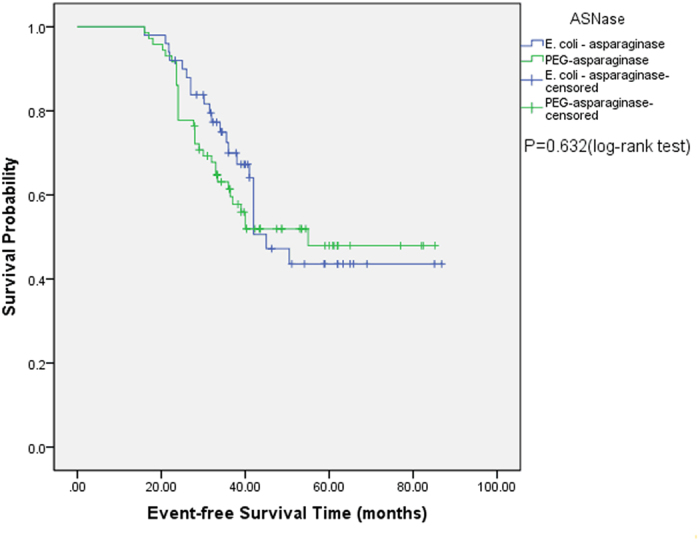
Comparison of probability of EFS between the *E. coli-*asparaginase group and PEG-asparaginase group. The predicted 5-year EFS was 43.6% and 46.9%, respectively. *P* = 0.632, log-rank test.

**Figure 2:**
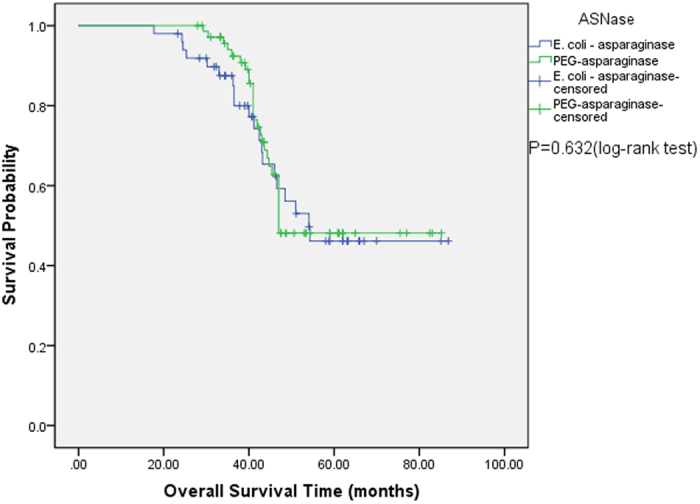
Comparison of probability of OS between the *E. coli-*asparaginase group and PEG-asparaginase group. The predicted 5-year OS was 46.2% and 48.1%, respectively. *P* = 0.769, log-rank test.

Comparison of outcomes for the *E. coli*-asparaginase and PEG-asparaginase groups is presented in Table 4. In total, 22 patients (44.0%) achieved early CR on day 14 in the *E. coli*-asparaginase group, whereas 48 (66.7%) patients achieved early CR in the PEG-asparaginase group (*P* = 0.016), indicating a more rapid clearance of blasts on day 14 in the PEG-asparaginase arm. At a median follow-up of 41.2 (17.7–86.8) and 43.6 (18.4–85.2) months in *E. coli*-asparaginase and PEG-asparaginase groups from the treatment commencement, respectively, no significant difference was observed in 5-year EFS: 46.9% for PEG-asparaginase group versus 43.6% for *E. coli*-asparaginase group (*P* = 0.632) (Fig. 1). Similarly, no difference was observed when comparing 5-year OS (48.1% and 46.2% for PEG-asparaginase and *E. coli*-asparaginase groups, respectively (*P* = 0.769) (Fig. 2)), despite the superior early response of induction therapy with PEG-asparaginase (increased CR frequency on day 14 during induction 1).

Since January 2009, minimal residual disease (MRD) by multiparameter flow cytometry (MFC) was performed on remission bone marrow specimens at the time of achievement of CR and then every cycle of therapy. In total, 69 patients were evaluable in the MRD assessment (29 in *E. coli*-asparaginase group and 40 in PEG-asparaginase group). In the *E. coli*-asparaginase group, MRD negativity was noted in 22 (75.9%) patients after 1 cycle and 24 (82.8%) patients after 2 or more cycles. In addition, MRD negativity was achieved by 30 (75.0%) patients after 1 cycle and 33 (82.5%) patients after 2 or more cycles in the PEG-asparaginase group. For the patients evaluable in the MRD assessment, MRD at the end of cycle 1 had a significant impact on survival (data not shown). Due to inadequate MRD data, additional survival analysis of MRD in the total cohort was not conducted.

Relapses were observed in 22/50 (44.0%) in the *E. coli*-asparaginase group and 33/72 (45.8%) in the PEG-asparaginase group (*P* = 0.856). Among the relapsing patients in the *E. coli*-asparaginase group, 12/22 (54.5%) were able to achieve a second CR (CR2) after re-induction chemotherapy, and 10 patients in CR2 were subsequently able to proceed to allo-HSCT. In comparison, for the 33 relapsing patients in the PEG-asparaginase group, 15/33 (45.5%) achieved CR2, and 11 patients in CR2 went on allo-HSCT. The median OS of patients post-relapse was 13 months (range 2–31 months) in the *E. coli*-asparaginase group and 11 months (range 2–32 months) in the PEG-asparaginase group.

### Prognostic factors

Table 5 displays the results of the univariate and multivariate analyses of the potential predictors of EFS and OS. Multivariate analysis using the forward conditional Cox region model identified later CR (relative risk = 2.082, 95% CI 1.208–3.587, *P* = 0.008) and older age (relative risk = 1.758, 95% CI 1.003–3.083, *P* = 0.049) as two adverse factors for EFS. Similarly, in the multivariate analysis for OS, later CR (relative risk = 1.964, 95% CI 1.082–3.564, *P* = 0.026) and older age (relative risk = 2.208, 95% CI 1.226–3.976, *P* = 0.008) were also identified as significant independent worse predictors of OS.

## Discussion

Currently, approximately 50% of adults with standard-risk ALL are cured with modern intensive chemotherapy that continues for 2 to 3 years of total therapy. Late treatment-related complications are uncommon after chemotherapy alone. Thus, the chronic disease burdens and diminished quality of life associated with allo-HSCT can be avoided. In addition, monitoring for MRD may identify a selected subset of ALL patients in CR1 who are likely to relapse and would most likely benefit from an early allo-HSCT^30^. Allo-HSCT can rescue some patients in CR2. Hence, chemotherapy with intensification, consolidation, and maintenance rather than allo-HSCT is recommended for adults with standard-risk ALL after CR1 at our department.

The implementation of regimens in adults with ALL has not been well studied, particularly for asparaginase, which is standard in children. *E. coli*-asparaginase and PEG-asparaginase have major differences in pharmacokinetic parameters and immunogenicity^4^,^13^. To date, no studies have compared *E. coli*-asparaginase with PEG-asparaginase based on their outcome and toxicities in adults with standard-risk ALL. Therefore, we conducted this retrospective study to evaluate the efficacy, safety, and toxicity of *E. coli*-asparaginase and PEG-asparaginase in these patients.

Of all the pharmacokinetic data, perhaps the most important characteristic of the PEG-asparaginase is the prolonged half-life. PEG-asparaginase has a t_1/2_ of approximately 6 days, which is far longer than that of the *E. coli*-asparaginase. Doses of PEG-asparaginase that have been used in ALL are 2000 to 2500 IU/m^2^, and dosing intervals ranged within 2 weeks^31^,^32^. Several initial studies suggested that the present dose and substitution used is 2000 to 2500 IU/m^2^ for each series of 6 to 9 doses of *E. coli*-asparaginase^33^. Thus, we used 3 doses of PEG-asparaginase at 2000 IU/m^2^ instead of 18 doses of *E. coli*-asparaginase at intervals of 2 weeks. Previous studies indicated that most patients with *E. coli*-asparaginase allergy also have a neutralizing antibody to the enzyme, resulting in sub-therapeutic systemic asparaginase activity even if allergic symptoms are prevented by the use of antihistamines and other premedications^34^,^35^,^36^,^37^,^38^. *Erwinia* asparaginase is an alternative preparation that is antigenically distinct from *E. coli-*asparaginase and PEG-asparaginase and has been administered to patients who experienced allergy to *E. coli-*asparaginase and PEG-asparaginase^39^,^40^. Thus, the development of allergy to *E. coli-*asparaginase required a change to *Erwinia* asparaginase in our department.

No TRM event occurred in the *E. coli*-asparaginase group, and 3 TRM events occurred in the PEG-asparaginase group without relation to asparaginase. In addition, we monitored the known adverse effects of asparaginase, and no asparaginase-related deaths were observed. A total of 9 patients using *E. coli-*asparaginase had grade 1/2 allergic reactions, whereas no patient using PEG-asparaginase experienced allergic reactions. The incidence of allergy was increased in the *E. coli*-asparaginase group compared with the PEG-asparaginase group with statistical significance (*P* < 0.001).

In our present retrospective study, no grade 1/2 pancreatitis was observed, and the incidence of grade 3/4 pancreatitis in the two groups was 9.8% vs. 5.8%, *P* = 0.715 (Table 3), which appeared to be consistent with some other studies with adult ALL^17^,^41^ and no greater than the value observed in pediatric patients^3^,^4^,^6^. Although three decades have elapsed since asparaginase-related pancreatitis was first described, the mechanism of this toxicity remains unknown^42^.

No statistical significance was observed in the incidence of hyperglycemia and hypertriglyceridemia in either grade 1/2 or grade 3/4 (Table 3). The incidence rates of hyperglycemia and hypertriglyceridemia in both groups were also similar with some previous studies^17^,^43^,^44^. Furthermore, two endocrine complications were commonly observed in induction regimen (data not shown), which included a large dose of prednisone. Similarly, the concomitant use of both drugs synergistically increases the occurrence of hyperglycemia and hypertriglyceridemia^45^.

The most common toxicities were hepatic (hyperbilirubinemia, transaminitis, hypoalbuminemia, and hypofibrinogenemia). No differences were observed regarding the incidence of these hepatic toxicities between the two groups (Table 3). The mechanism of asparaginase-related hepatotoxicity is hypothesized to be caused by the declined protein synthesis or impaired liver mitochondrial function^46^,^47^. However, determining the reasons for liver toxicity when the underlying disease, infection, co-morbidities, and other hepatotoxic agents can all be inciting factors is difficult. As recommended by other clinical experiences and the literature to date^48^,^49^, we would delay but not reduce the dose of asparaginase when grade 3/4 hepatotoxicity developed and subsequently rechallenge with careful monitoring when toxicity resolves to grade 1/2 because the injury to the liver itself is transient. Conservative use of cryoprecipitate was administered when serum fibrinogen was less than 50 mg/dL.

Thrombosis was uncommon in our study, and all the thrombotic events were related to venous catheters. The incidence was lower than some previous reports on the toxicity of asparaginase^17^,^50^,^51^, which may be explained by avoiding routinely infusing cryoprecipitate only when fibrinogen level was less than 50 mg/dL, according to the recommendation from the French CAPELAL study^51^. Routine administration of cryoprecipitate was not beneficial in preventing thrombosis in a pediatric study^52^ because cryoprecipitate contains high concentrations of factor VIII and is therefore particularly thrombogenic. No clear conclusion is available in the literature regarding whether further administration of asparaginase should be terminated when thrombosis occurs in adults. Asparaginase was continued after effective anticoagulation without recurrence of complications in the CAPELAL study^51^. Thus, we continued the use of asparaginase with anticoagulation therapy and closely monitored the patients when thrombosis occurred.

Importantly, statistically significant superiority was documented for PEG-asparaginase compared with *E. coli*-asparaginase in terms of the clearance of lymphoblasts from day 14 bone marrow aspirates in our adults. The reason for the difference is unknown but could be from persistent, high asparaginase activity in the PEG-asparaginase patients. However, our study is not sufficient to detect a superior EFS or OS in the PEG-asparaginase group. At a median follow-up of 43.6 months in the PEG-asparaginase group, a 5-year EFS rate of 46.9% and a 5-year OS rate of 48.1% were observed. Moreover, 5-year EFS and 5-year OS rates of 43.6% and 46.2%, respectively, were observed in the *E. coli-*asparaginase group, with a median follow-up of 41.2 months. Our survival data were similar to the data for other standard-risk ALL patients (only receiving chemotherapy after CR1) from several clinical trials in which no significant benefit for allo-HCT was observed compared with chemotherapy with respect to EFS and OS among standard-risk ALL patients^53^,^54^,^55^,^56^.

We also conducted univariate and multivariate analyses of the potential predictors of EFS and OS in adults with standard-risk ALL. Later CR and older age were suggested as adverse independent prognostic factors. Early response to treatment is one of the most important prognostic factors in children with ALL, which was assessed by day 7 and 14 bone marrow morphology or day 7 peripheral blast count^6^. This conclusion also applies equally to adults with standard-risk ALL. Age is also one of the most important prognostic factors in the total cohort of ALL because the prevalence of high-risk biological leukemia (e.g., *BCR-ABL* and *MLL* rearrangements) and unfavorable factors, such as high WBC count, increase with age. Nevertheless, in the absence of the known risk and unfavourable factors mentioned above, age can also indicate prognosis in adults with standard-risk ALL, which may be explained by the poor adherence to and tolerance of therapy or other unknown worse biological factors in the older subgroup.

As mentioned above, early CR was associated with improved EFS and OS upon multivariate analysis. Patients using PEG-asparaginase achieved a higher early CR rate that did not transform into superior EFS or OS. Various reasons may contribute to the inconsistency. Our study was a retrospective study in a single center with a small sample size. No TRM event occurred in the *E. coli*-asparaginase group, whereas 3 TRM events occurred without relapse in PEG-asparaginase group. The follow-up treatments after relapse were not comparable and were performed at the discretion of the treating physician on the basis of donor availability.

MRD by MFC was performed since January 2009. In total, 69 patients received MRD assessment. In these patients mentioned above, MRD at the end of cycle 1 had a significant impact on survival. Due to inadequate MRD data for all patients, a comparison of MRD status between the two groups was not conducted. In addition, MRD was not evaluated in the multivariate analysis. Further efforts are still needed to develop a precise risk stratification including MRD to distinguish patients who need allo-HSCT treatment in CR1.

Several limitations in our present analysis are discussed below. It is important to note that this was a retrospective study in a single center, and the sample size was small. Moreover, due to the retrospective character of this analysis, the monitoring of toxicities might not be standard in some patients and toxicities were likely underestimated. In addition, we have no laboratory data on the monitoring of asparaginase trough levels and effective serum asparagine depletion from our patients treated with PEG-asparaginase and *E. coli-*asparaginase. Future prospective randomized clinical trials may be warranted to compare the efficacy and safety of the two asparaginase preparations and to optimize the dose and the frequency and route of administration of PEG-asparaginase in adults with newly diagnosed ALL.

In conclusion, PEG-asparaginase administered in the context of the UKALLXII/ECOG E2993-based chemotherapy regimen at 2000 IU/m^2^ per dose is safe for adults with standard-risk ALL, and substitution of PEG-asparaginase for *E. coli*-asparaginase during induction and intensification conferred a statistically significant advantage in the CR rate on day 14 but was not sufficient to detect a difference in the EFS and OS. Therefore, PEG-asparaginase may have the same efficacy as *E. coli-*asparaginase and be safely used as another choice of asparaginase in adults with standard-risk ALL given its more rapid clearance of lymphoblasts on day 14, reduced incidence of allergy, and similar long-term outcome and convenience.

## Methods

### Patients

This study was a retrospective study that involved adult patients with newly diagnosed standard-risk ALL defined according to the UKALLXII/ECOG E2993 protocol and satisfying the following criteria^28^: Philadelphia chromosome, negative; age, ≤35 years; time taken to achieve CR, ≤4 weeks; WBC count, <30 × 10^9^/L for B-lineage ALL and <100 × 10^9^/L for T-lineage ALL, excluding t(4;11) *MLL* translocation, complex karyotype (≥5 chromosomal abnormalities), and low hypodiploidy (30–39 chromosomes)/near triploidy (60–78 chromosomes). Patients were treated with induction, intensification, consolidation, and maintenance regimens at the Department of Hematologic Oncology, Cancer Center, Sun Yat-sen University between January 2005 and January 2013. All patients were diagnosed on the basis of clinical features, bone marrow cytology, flow cytometric evaluation, and cytogenetic and molecular biology detection. Pertinent data were obtained from clinical files and the electronic database.

The study was approved by the Ethical committee of Sun Yat-sen University Cancer Center, and written informed consent was provided by the patients. All experiments were performed in accordance with relevant guidelines and regulations.

### Treatment

Patients included for analysis received phase 1 of induction therapy. Bone marrow aspirates were conducted at entry and on day 14 and day 28 of induction 1. Patients who did not achieve CR on day 28 were excluded. Included patients went on to phase 2 of induction, intensification, consolidation, and maintenance using the UKALLXII/ECOG E2993-based regimen^28^ incorporating 18 doses of *E. coli-*asparaginase or 3 doses of PEG-asparaginase into the chemotherapy of induction and intensification, respectively (Table 1). The development of allergy to *E. coli-*asparaginase or PEG-asparaginase required a change to *Erwinia* asparaginase, and the patients were excluded.

Regarding relapsed patients, second-line chemotherapy regimens and allo-HSCT were performed at the discretion of the treating physician and on the basis of donor availability.

### Toxicity and laboratory monitoring

TRM was defined as death within 30 days or during hospitalization. Toxicities during each cycle were abstracted by a single investigator from the biochemistry panel results (at least once weekly) and diagnoses in the medical chart. Toxicities were graded prospectively according to the National Cancer Institute Common Terminology Criteria for Adverse Events version 4.0. Specific events of interest were allergy, pancreatitis, transaminitis, hyperbilirubinemia, hypoalbuminemia, hyperglycemia, hypertriglyceridemia, hypofibrinogenemia, and DVT during the asparaginase-containing phases of chemotherapy.

### Response criteria and MRD assessment

Patients were considered in CR when bone marrow cellularity was greater than 20% with maturation of all cell lines and less than 5% blasts. Granulocyte count was greater than 1.5 × 10^9^/L, platelet count was greater than 100 × 10^9^/L, leukemic blasts were absent from the peripheral blood, and any detectable extramedullary leukemia had resolved. Isolated and stable splenomegaly was not considered extramedullary disease.

Since January 2009, MRD by MFC was performed on remission bone marrow specimens at the time of achievement of CR and then every cycle of therapy as previously described^57^. Initially, a 15-marker, 6-color panel was used; later, an 8-color panel was used. MRD positivity was defined on MFC scatter plots as a cluster of at least 20 cells exhibiting altered expression of two or more antigens. The sensitivity of this MRD assay is 0.01%.

### Statistical analysis

Statistical group comparisons of categorical variables were performed using the χ2 test/Fisher’s exact test, and quantitative data, such as age and WBC count, were analysed using the non-parametric Mann–Whitney *U*-test test. Analysis of clinical outcomes included early response rates during induction at day 14 and relapse rate. We also examined the OS and EFS. OS was calculated from the time of diagnosis of death from any cause. EFS was the time to defined events, death, or recurrence of disease. Kaplan–Meier estimates were computed for OS and EFS. For patients without an event, observation was censored at the last contact date. The Kaplan–Meier method and log-rank test were also applied for univariate analyses of the impacts of several clinical characteristics on survival. Variables, which were significant at *P* < 0.05 in univariate analysis, were used in the multivariate analysis. Multivariate analysis was used to assess the prognostic impact of selected variables according to the Cox regression model. Statistical analysis was performed using the SPPS statistical package (SPPS V21.0).

## Additional Information

**How to cite this article**: Liu, W.-J. *et al*. Use of PEG-asparaginase in newly diagnosed adults with standard-risk acute lymphoblastic leukemia compared with *E. coli*-asparaginase: a retrospective single-center study. *Sci. Rep.* **6**, 39463; doi: 10.1038/srep39463 (2016).

**Publisher's note:** Springer Nature remains neutral with regard to jurisdictional claims in published maps and institutional affiliations.

## Figures and Tables

**Table 1:** Treatment schema for *E. coli-*asparaginase and PEG-asparaginase groups.

*E. coli-*asparaginase group		PEG-asparaginase group	
Therapy	Dosage and days	Therapy	Dosage and days
**Induction**			
Phase 1			
Daunorubicin IV	60 mg/m^2^ days 1, 8, 15, 22	Daunorubicin IV	60 mg/m^2^ days 1, 8, 15, 22
Vincristine IV	1.4 mg/m^2^ days 1, 8, 15, 22	Vincristine IV	1.4 mg/m^2^ days 1, 8, 15, 22
*E. coli-*asparaginase IV	10000 IU qod days 2–24 (12 doses)	PEG-asparaginase IM	2000 IU/m^2^ days 2, 16
Prednisone PO	60 mg/m^2^ days 1–28	Prednisone PO	60 mg/m^2^ days 1–28
Methotrexate IT	12.5 mg day 15	Methotrexate IT	12.5 mg day 15
Phase 2			
Cyclophosphamide IV	650 mg/m^2^ days 1, 15, 29	Cyclophosphamide IV	650 mg/m^2^ days 1, 15, 29
Cytarabine IV	75 mg/m^2^ days 1–4, 8–11, 15–18, 22–25	Cytarabine IV	75 mg/m^2^ days 1–4, 8–11, 15–18, 22–25
6-mercaptopurine PO	6 mg/m^2^ days 1–28	6-mercaptopurine PO	6 mg/m^2^ days 1–28
Methotrexate IT	12.5 mg day 1, 8, 15, 22	Methotrexate IT	12.5 mg day 1, 8, 15, 22
Intensification			
Methotrexate IV	3 g/m^2^ days 1, 8, 22	Methotrexate IV	3 g/m^2^ days 1, 8, 22
*E. coli-*asparaginase IV	10000 IU qod days 2–12 (6 doses)	PEG-asparaginase IM	2000 IU/m^2^ day 2
**Consolidation**			
Cycle 1			
Cytarabine IV	75 mg/m^2^ days 1–5	Cytarabine IV	75 mg/m^2^ days 1–5
Etoposide IV	100 mg/m^2^ days 1–5	Etoposide IV	100 mg/m^2^ days 1–5
Vincristine IV	1.4 mg/m^2^ days 1, 8, 15, 22	Vincristine IV	1.4 mg/m^2^ days 1, 8, 15, 22
Dexamethasone PO	10 mg/m^2^ days 1–28	Dexamethasone PO	10 mg/m^2^ days 1–28
Cycle 2			
Cytarabine IV	75 mg/m^2^ days 1–5	Cytarabine IV	75 mg/m^2^ days 1–5
Etoposide IV	100 mg/m^2^ days 1–5	Etoposide IV	100 mg/m^2^ days 1–5
Cycle 3			
Daunorubicin IV	25 mg/m^2^ days 1, 8, 15, 22	Daunorubicin IV	25 mg/m^2^ days 1, 8, 15, 22
Cyclophosphamide IV	650 mg/m^2^ day 29	Cyclophosphamide IV	650 mg/m^2^ day 29
Cytarabine IV	75 mg/m^2^ days 31–34, 38–41	Cytarabine IV	75 mg/m^2^ days 31–34, 38–41
Thioguanine PO	60 mg/m^2^ days 29–42	Thioguanine PO	60 mg/m^2^ days 29–42
Cycle 4			
Cytarabine IV	75 mg/m^2^ days 1–5	Cytarabine IV	75 mg/m^2^ days 1–5
Etoposide IV	100 mg/m^2^ days 1–5	Etoposide IV	100 mg/m^2^ days 1–5
CNS prophylaxis in consolidation: Cytarabine IT, 50 mg, was given weekly for 4 weeks, together with 2400 cGy cranial irradiation			
Maintenance (2.5 years from the start of intensification therapy)			
Vincristine IV	1.4 mg/m^2^ day 1*every 3 months	Vincristine IV	1.4 mg/m^2^ day 1* every 3 months
Prednisone PO	60 mg/m^2^ days 1–5* every 3 months	Prednisone PO	60 mg/m^2^ days 1–5* every 3 months
6-mercaptopurine PO	75 mg/m^2^*qd	6-mercaptopurine PO	75 mg/m^2^*qd
Methotrexate PO	20 mg/m^2^*qw	Methotrexate PO	20 mg/m^2^*qw
CNS prophylaxis in maintenance: Cytarabine IT, 50 mg, was given 3 months apart during maintenance therapy.			

Abbreviations: *E. coli-*asparaginase, *Escherichia coli* asparaginase; PEG-asparaginase, polyethylene glycol-conjugated asparaginase; IV, intravenously; PO, oral; IM, intramuscular; IT, intrathecal.

**Table 2:** Distribution of patient characteristics by treatment assignment.

	*E. coli-*asparaginase (n = 50)	PEG-asparaginase (n = 72)
Median age (range), years	27 (18–35)	26 (18–35)
WBC, median (range) × 10^9^/L	26.25 (0.50–94.06)	26.92 (1.08–99.74)
Age, n (%)		
18–26 years	24 (48.0)	37 (51.4)
27–35 years	26 (52.0)	35 (48.6)
Sex, n (%)		
Male	37 (74.0)	47 (65.3)
Female	13 (26.0)	25 (34.7)
Immunophenotype, n (%)		
B	37 (74.0)	49 (68.1)
T	13 (26.0)	23 (31.9)
ECOG performance status, n (%)		
0–1 scores	38 (76.0)	57 (79.2)
≥ 2 scores	12 (24.0)	15 (20.8)
CSF, n (%)		
Positive	0 (0.0)	0 (0.0)
Negative	50 (100.0)	72 (100.0)
Organ involvement, n (%)		
Mediastinal mass	11 (22.0)	16 (22.2)
Hepatomegaly	7 (14.0)	11 (15.3)
Splenomegaly	6 (12.0)	14 (19.4)
Lymphadenopathy	10 (20.0)	13 (18.1)

Abbreviations: *E. coli-*asparaginase, *Escherichia coli* asparaginase; PEG-asparaginase, polyethylene glycol-conjugated asparaginase; WBC, white blood cell; ECOG, Eastern Cooperative Oncology Group; CSF, cerebrospinal fluid.

**Table 3:** Toxicity in the *E. coli-*asparaginase and PEG-asparaginase groups.

	Grades 1/2 n (%)		*P*	Grades 3/4 n (%)		*P*
	*E. coli-* asparaginase	PEG- asparaginase		*E. coli-* asparaginase	PEG- asparaginase	
Pancreatitis	0 (0.0)	0 (0.0)	—	4 (9.8)	4 (5.8)	0.715
Transaminitis	11 (21.6)	13 (30.8)	0.647	7 (15.7)	8 (13.5)	0.780
Hyperbilirubinemia	13 (15.7)	15 (19.2)	0.519	8 (21.6)	13 (13.5)	0.812
Hypoalbuminemia	11 (25.5)	14 (28.8)	0.821	8 (19.6)	9 (21.2)	0.604
Hyperglycemia	6 (15.7)	11 (21.2)	0.791	8 (15.7)	13 (9.6)	0.812
Hypertriglyceridemia	7 (17.6)	12 (23.1)	0.802	5 (13.7)	10 (15.4)	0.586
Hypofibrinogenemia	11 (25.5)	18 (21.2)	0.830	8 (13.7)	10 (23.1)	0.798
DVT	1 (2.0)	4 (2.0)	0.648	3 (5.9)	2 (1.9)	0.399

Abbreviations: *E. coli-*asparaginase, *Escherichia coli* asparaginase; PEG-asparaginase, polyethylene glycol-conjugated asparaginase; DVT, deep vein thrombosis.

**Table 4:** Outcomes by treatment received.

	*E. coli-*asparaginase (n = 50)	PEG-asparaginase (n = 72)	*P*
CR on day 14, n (%)	22 (44.0)	48 (66.7)	0.016
Relapse, n (%)	22 (44.0)	33 (45.8)	0.856
5-year EFS rate (%)	43.6	46.9	0.632
5-year OS rate (%)	46.2	48.1	0.769

Abbreviations: *E. coli-*asparaginase, *Escherichia coli* asparaginase; PEG-asparaginase, polyethylene glycol-conjugated asparaginase; CR, complete remission; EFS, event-free survival; OS, overall survival.

**Table 5:** Univariate and multivariate analysis of factors associated with EFS and OS of all patients.

Clinical characteristics	Event-free survival			Overall survival		
	Univariate analysis	Multivariate analysis		Univariate analysis	Multivariate analysis	
	*P*	RR (95% CI)	*P*	*P*	RR (95% CI)	*P*
Gender (female/male)	0.868			0.804		
Age (27–35/18–26 years)	0.025	1.758 (1.003–3.083)	0.049	0.007	2.208 (1.226–3.976)	0.008
Immunophenotype (B/T)	0.470			0.894		
Asparaginase (*E. coli-*asparaginase/PEG-asparaginase)	0.632			0.769		
ECOG performance status (≥2/0–1 scores)	0.014	1.679 (0.922–3.059)	0.090	0.092		
Mediastinal mass	0.645			0.589		
Hepatomegaly	0.017	1.052 (0.177–6.235)	0.956	<0.001	1.510 (0.237–9.602)	0.662
Splenomegaly	0.027	1.933 (0.345–10.837)	0.454	0.002	1.907 (0.318–11.441)	0.480
Lymphadenopathy	0.720			0.741		
CR on (day28/day14)	0.003	2.082 (1.208–3.587)	0.008	0.004	1.964 (1.082–3.564)	0.026

Abbreviations: *E. coli-*asparaginase, *Escherichia coli* asparaginase; PEG-asparaginase, polyethylene glycol-conjugated asparaginase; CR, complete remission; RR, relative risk. Table.
